# Shifting the Balance: How Top-Down and Bottom-Up Input Modulate Pain *via* the Rostral Ventromedial Medulla

**DOI:** 10.3389/fpain.2022.932476

**Published:** 2022-06-28

**Authors:** Qiliang Chen, Mary M. Heinricher

**Affiliations:** ^1^Department of Anesthesiology, Perioperative and Pain Medicine, Stanford University, Stanford, CA, United States; ^2^Department of Neurological Surgery and Behavioral Neuroscience, Oregon Health & Science University, Portland, OR, United States

**Keywords:** pain-modulation, descending control, brainstem, analgesia, hyperalgesia, rostral ventromedial medulla, RVM

## Abstract

The sensory experience of pain depends not only on the transmission of noxious information (nociception), but on the state of the body in a biological, psychological, and social milieu. A brainstem pain-modulating system with its output node in the rostral ventromedial medulla (RVM) can regulate the threshold and gain for nociceptive transmission. This review considers the current understanding of how RVM pain-modulating neurons, namely ON-cells and OFF-cells, are engaged by “top-down” cognitive and emotional factors, as well as by “bottom-up” sensory inputs, to enhance or suppress pain.

## Introduction

Pain is an unpleasant sensory and affective experience associated with actual or potential tissue damage, serving as a survival mechanism that triggers escape from injurious or potentially injurious events, promotes recuperative behaviors, and motivates learning that leads to avoidance of such stimuli in the future. The neural system encoding and processing noxious or potentially harmful stimuli is termed “*nociception*.” However, *pain*, a sensory experience, is not a direct readout of nociceptive inputs. Indeed, pain associated with a given stimulus varies quite strongly with context and behavioral state.

A dramatic example of the gap between nociception and pain is “stress-induced analgesia,” which allows an organism to escape from an immediate threat, e.g., a predator, even in the face of injuries that would normally evoke significant pain (1–3). Stress-induced analgesia prevents pain behaviors, such as tending to an injured extremity, that could interfere with an escape from the threat (4). At the opposite end of the spectrum, pain can be enhanced by stress that is less intense or by anxiety. In a state of “stress-induced hyperalgesia,” innocuous inputs may be perceived as painful (5, 6). Both stress-induced analgesia and stress-induced hyperalgesia have been demonstrated in humans and can be seen outside of the artificial environment of the laboratory, for example after severe trauma (3, 7–10). More broadly, there is abundant evidence that pain is influenced by cognitive and emotional factors, and that it can vary, subtly or dramatically, with context and behavioral state (11–13).

This flexibility in the experience of pain arises from plasticity and modulation of multiple neural circuits. This can include changes in sensitivity of primary sensory neurons, alterations in the function of nociceptive neurons at spinal and supraspinal levels, and engagement of specific pain-modulating systems. Here we focus on the latter, and specifically on the rostral ventromedial medulla (RVM), the output node of a complex pain-modulating system. The RVM projects to the dorsal horn where it controls the processing and transmission of nociceptive information, which in turn modifies the ascending signal reaching the brain (14, 15). The present review will emphasize recent findings revealing how this pain-modulating system, and specifically the RVM, can be brought into play by both “top-down” (cognitive, emotional) and “bottom-up” (sensory) inputs to enhance or suppress pain.

## Rostral Ventromedial Medulla (RVM)

**Figure 1:**
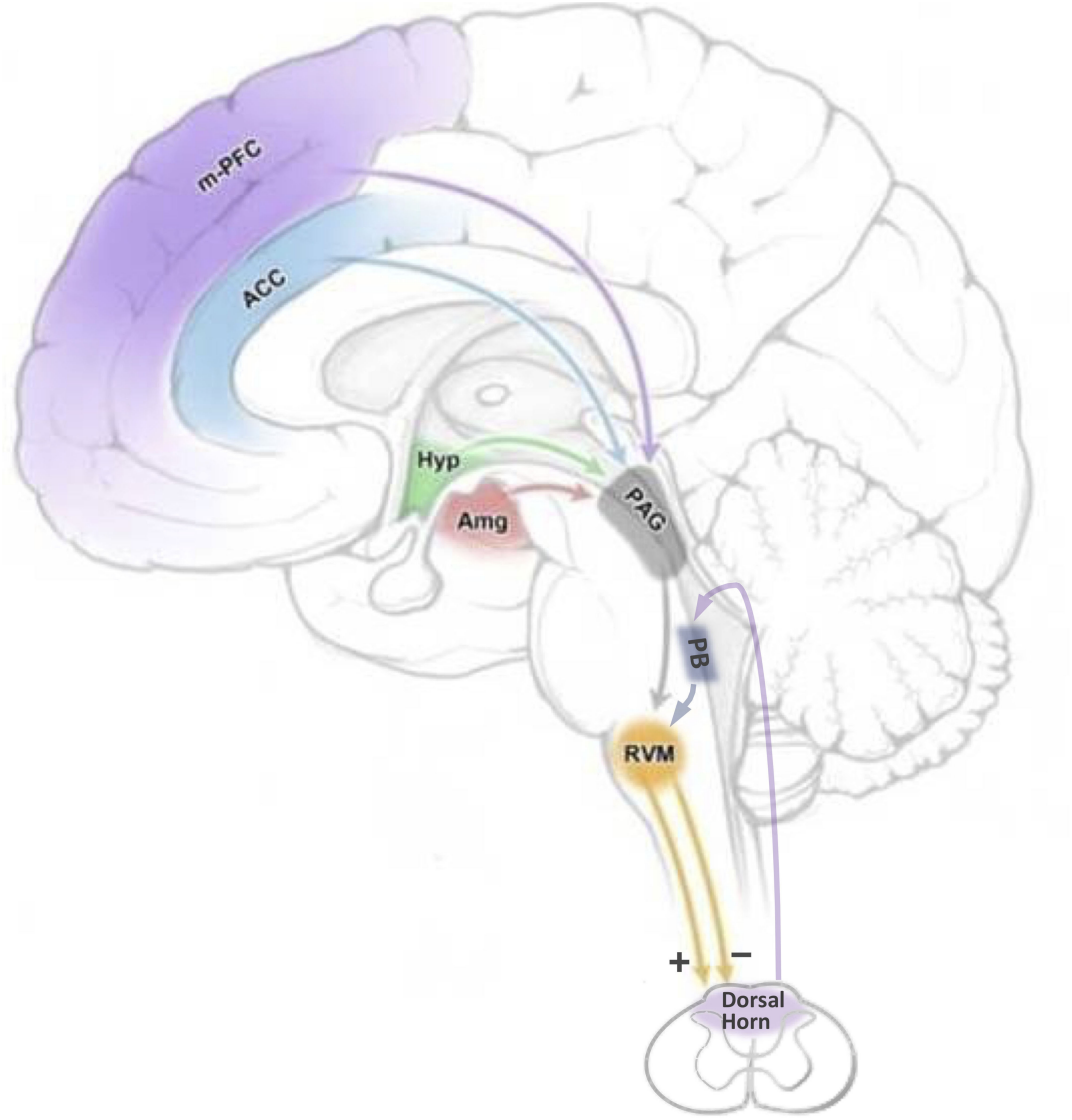
Top-down and bottom-up inputs through which cognitive and emotional factors could influence nociceptive transmission by modulating the firing of RVM pain-modulating neurons. Cognitive and emotional processes mediated by higher structures have the potential to influence the RVM through the PAG, and in some cases, directly. Bottom-up, nociceptive, inputs are relayed from the spinal cord to the RVM *via* the parabrachial complex. ACC: anterior cingulate cortex; Amg: amygdala; Hyp: hypothalamus; m-PFC: medial prefrontal cortex; PAG: periaqueductal gray; PB: parabrachial complex; RVM: rostral ventromedial medulla. Adapted from Cleary, D. and Heinricher, M.M. Neuroanatomy and neurophysiology of pain. In Winn, H.R. (Ed)., *Youmans and Winn Neurological Surgery, 8th Ed*., Elsevier Saunders, Philadelphia, 2022.

The RVM is defined functionally, as an area over which low-intensity ( ≤ 10 μA) electrical stimulation produces profound antinociception (18). Anatomically, the RVM is not only centered on the nucleus raphe magnus but also includes adjacent ventromedial reticular formation. The positioning of the RVM as the output node of a complex, brain-spanning network is schematized in Figure 1. The RVM receives top-down input from higher structures such as the amygdala, hypothalamus, infralimbic and prelimbic cortex, and insula, both directly and indirectly *via* the midbrain periaqueductal gray (PAG). The RVM also receives bottom-up input, including nociceptive information, which allows ongoing or recent noxious stimuli to influence the response to a new insult (17, 19, 20). The primary output of the RVM is to the spinal and trigeminal dorsal horns, where it regulates nociceptive processing. Importantly, this modulation is not limited to spinally mediated manifestations of nociception such as withdrawal reflexes; because it controls ascending transmission, it influences both affective and sensory dimensions of pain (21–23).

Based on early studies using electrical stimulation to produce analgesia, the RVM was originally viewed as part of a descending pain-inhibitory circuit or “analgesia system” (24, 25). This point of view was reinforced by evidence that the analgesic effects of mu-opioid agonists are at least partly mediated by direct actions in the RVM (26–28). However, it subsequently became clear that the RVM can also *enhance* nociception, and that it functions to facilitate, as well as inhibit, pain. The facilitating output from the RVM has been implicated in hypersensitivity and spontaneous pain in a wide range of chronic pain models (22, 23, 29, 30).

### The Antinociceptive and Pronociceptive Outputs From the RVM Are Mediated by Distinct, Physiologically Definable, Classes of Neurons

**Figure 2:**
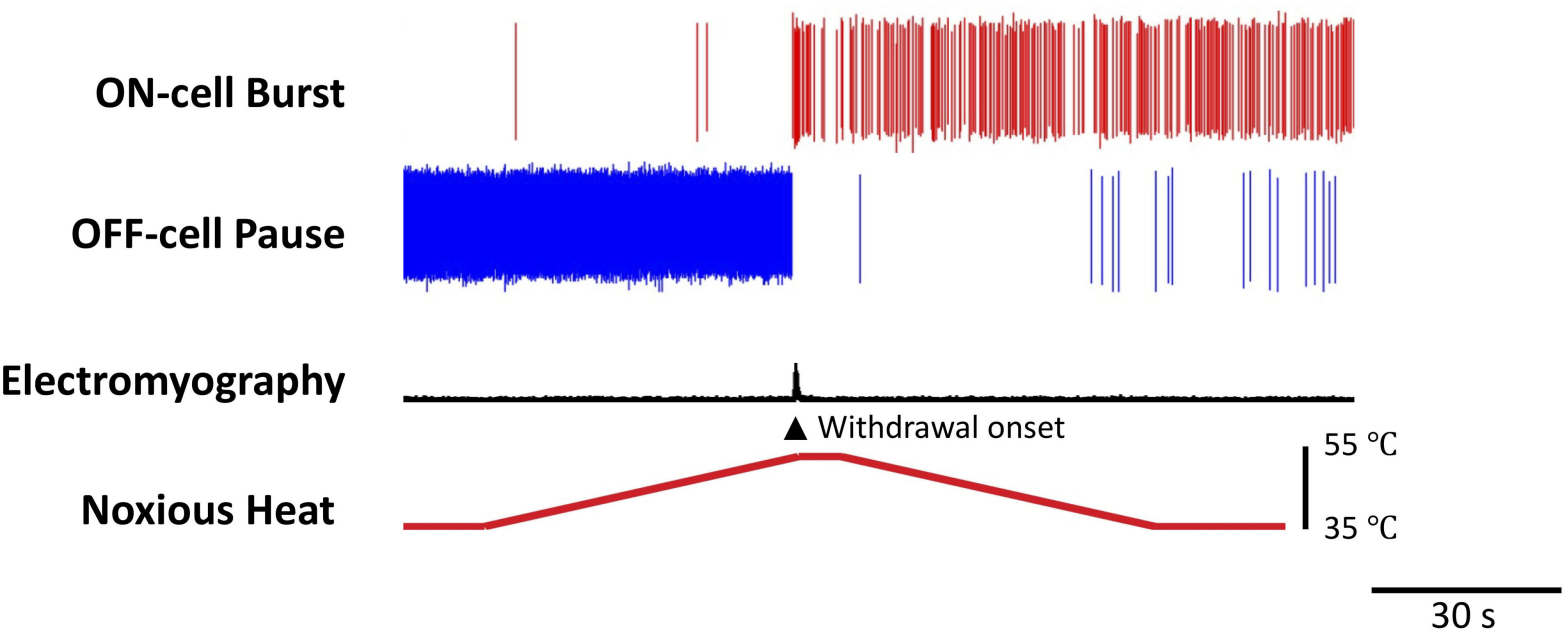
Reciprocal firing patterns of pain-modulating RVM ON- and OFF-cells. Examples of ON- and OFF-cell firing changes associated with nocifensive withdrawal evoked by a noxious heat stimulus. The OFF-cell pauses and ON-cell bursts immediately prior to the behavior. The threshold at which the OFF-cells pause correlates with the threshold for nocifensor withdrawals and periods of OFF-cell inactivity are associated with a lower threshold (16). In contrast, the magnitude of the ON-cell burst is positively correlated with the magnitude of behavioral responses (17). Heat (bottom trace) was applied to the plantar surface of the hind paw using a Peltier device. Withdrawal reflex was monitored using an electromyogram (EMG). The OFF-cell “pauses” and ON-cell “bursts” immediately prior to the withdrawal. The duration of the pause and burst can range from a few seconds, as here, to many minutes.

The pain-inhibiting and pain-facilitating outputs of the RVM are respectively mediated by two, physiologically defined, classes of neurons, referred to as “OFF-cells” and “ON-cells” [Figure 2, for historical review, see Fields and Heinricher (18)]. Pain-inhibiting OFF-cells exhibit a GABA-mediated “pause” in any ongoing firing beginning a few hundred milliseconds prior to nocifensive withdrawal behaviors, and the block of that pause produces antinociception. ON-cells enter an active state (“burst”) beginning just after the OFF-cells stop firing and immediately prior to a nocifensive withdrawal. ON-cell activity enhances nociception, and selective activation of ON-cells (e.g., pharmacologically) is sufficient to produce measurable behavioral hyperalgesia (31). Conversely, selective block of ON-cell activity can reverse hyperalgesia, including stress-induced hyperalgesia (32–37).

Not all RVM neurons are OFF-cells or ON-cells. However, the remaining cells, called “NEUTRAL-cells,” show no nociceptive responses and exhibit pharmacology distinct from that of ON- and OFF-cells. Although all three classes are output neurons, and project to the dorsal horn (38, 39), whether NEUTRAL-cells have a role in pain-modulation remains an open question.

ON-cells can be identified by expression of the mu-opioid receptor (40, 41), and express the neurokinin1 receptor (34). A molecular marker has not been identified as specific for either OFF-cells or NEUTRAL-cells, and it has become clear that none of the three classes maps to a particular neurotransmitter. For example, a majority of all three classes are GABAergic (42), and consistent with this, both pain-facilitating and pain-inhibiting outputs from the RVM have been shown to include GABAergic neurons in functional studies (43, 44). Serotonin is found in both ON-cells and NEUTRAL-cells (42, 45).

Both ON- and OFF-cells exhibit fluctuating spontaneous activity, with alternating silent and active periods. Activity within each class is in phase, and activity of the two classes is antiphase-synchronized under unstimulated conditions, as well as when an OFF-cell pause and ON-cell burst occur in association with a nocifensive withdrawal (46, 47). Thus, OFF-cells are silent when ON-cells are active and vice-versa. The complementary outputs from the two classes, therefore, modulate dorsal horn nociceptive transmission in parallel. Under basal conditions (i.e., in the absence of persistent injury or inflammation), the nociceptive threshold is measurably lower if a noxious stimulus is delivered during a period when the ON-cell population is active compared to when the OFF-cells are active (16), and inactivation of RVM can produce hyperalgesia due to loss of OFF-cell output (48). Subtle shifts in nociceptive sensitivity mediated by these normal fluctuations in ON- and OFF-cell firing likely contribute to the prioritization of pain behaviors relative to other behavioral priorities, such as feeding (49, 50). However, factors that eliminate the OFF-cell pause, such as mu-opioid agonist administration, can entirely suppress nociceptive behavior (51). Conversely, treatments that lead to sustained activation of ON-cells (and suppression of OFF-cell activity) produce hyperalgesia (31–33, 52–54). Interestingly, the net behavioral effect of experimental co-activation of OFF- and ON-cells (e.g., electrical stimulation or local administration of a high dose of a GABA_A_ receptor antagonist) is antinociception, implying that the antinociceptive effect of OFF-cell activity is sufficient to override the pro-nociceptive effect of ON-cell activity. However, these two classes are not generally simultaneously active in the absence of experimental manipulation.

Interactions within the RVM itself are almost entirely unexplored. One thing is known, however, which is that ON-cells are not inhibitory interneurons mediating the OFF-cell pause. This was originally seen as a possibility since the ON-cells are most active at the time of the OFF-cell pause (18). However, the inactivation of ON-cells has no effect on the OFF-cell pause (55), and the onset of the ON-cell burst almost invariably lags the start of the OFF-cell pause (56). These two lines of evidence argue strongly that the ON-cell burst and OFF-cell pause represent parallel processes, and that both are triggered by input from outside the RVM.

In sum, following the description of ON- and OFF-cells almost 40 years ago (47), the focus has been on how these neurons function as the *output* of a midline brainstem pain-modulating system. Experimental approaches using direct and selective activation or inactivation of each RVM cell class, as well as correlative analyses have demonstrated that OFF-cells exert a net antinociceptive effect and ON-cells a net pro-nociceptive effect. Moreover, a significant body of evidence points to a shift in the balance between OFF- and ON-cell activity such that ON-cells predominate as a factor in chronic pain (30, 57). This foundational work is now the basis for the next critical question: when and how is this system recruited to modulate pain? In the next sections, we consider “top-down” and “bottom-up” recruitment of the RVM to suppress or facilitate pain.

## “Top-Down” Engagement of Descending Control

### Pain Can Be Modulated by “Top-Down” Cognitive and Emotional Factors

The ability of “top-down” influences to modulate the experience of pain has been recognized for centuries. Stress-induced analgesia was an important stimulus to research in the latter half of the twentieth century: excitement was in part because delineation of the circuitry mediating stress-induced analgesia demonstrated the existence of an intrinsic capacity to modulate pain, and also because this same circuitry was discovered to mediate the analgesic effects of opioid analgesic drugs (11). However, modulation of pain by cognitive and emotional factors, including attention, placebo/nocebo, mood, social cues, and other motivational states is well documented, if generally less dramatic than stress-induced analgesia (12). Circuits mediating these effects are now a focus of research across the field, and cortico-cortical/cortico-limbic interactions almost certainly play some role. However, relevant cortical structures also have connections with the descending pain-modulation system (Figure 1). Anterior cingulate, prefrontal areas, and insula all project to the PAG and/or RVM, as do the amygdala and a number of hypothalamic nuclei (58–62). Moreover, there is evidence from imaging studies that the RVM is engaged in humans by a range of top-down factors, including attention and placebo (63–65). However, imaging studies, based on blood oxygenation, cannot distinguish recruitment of pain-facilitating ON-cells from activation of pain-suppressing OFF-cells, as these populations are not segregated anatomically. However, sophisticated studies using juxtacellular recording demonstrate that both ON-cells and OFF-cells receive inputs from the PAG, with the majority being GABAergic in both cases (NEUTRAL-cells also receive a substantial GABAergic projection from the PAG) (66). Below we consider some critical studies that tease out the recruitment of these defined pain-modulating populations by top-down influences.

### Fear and Stress Engage the RVM to Suppress or Enhance Pain

Intense fear or stress triggers *analgesia*, mediated by engagement of RVM OFF-cells *via* the basolateral amygdala (67, 68). By contrast, mild stress, such as air-puff to the face in rodents, can produce *hyperalgesia*. Inactivation of the dorsomedial hypothalamus (DMH) has been shown to interfere with a variety of stress-related responses, such as increased sympathetic drive and behavioral hyperactivity (69–71). DMH projects to the RVM, and stimulation of the DMH induces hyperalgesia mediated by RVM ON-cells, as well as physiological and behavioral changes associated with mild stress (32, 37, 72–74).

### Balance of RVM ON- and OFF-Cell Activity Is Modulated by Other Motivational States

The effect of behavioral state on pain is not limited to contexts with negative emotional valence. Feeding has been shown in several species to be accompanied by measurably reduced responses to noxious stimuli (49, 50, 75). This hypoalgesia is associated with reduced ON-cell activity and increased OFF-cell activity, and is eliminated by blockade of the RVM (75). However, the circuitry through which feeding-related input gains access to the RVM has not been determined.

Interestingly, there was a recent report that consumption of a sweet drink did not lead to hypoalgesia in adult humans, although sucrose is reported to ease pain in infants (76). These authors suggest that the lack of effect in adults may be because of the relative ease of access to sweets in modern society, reducing the hedonic impact of the manipulation. In any case, the idea that events with a positive hedonic valence can engage the RVM to modulate pain raises the question of how this happens, and tracing the relevant circuits is an interesting direction for future research. It should also be mentioned here that hunger, like feeding, has been reported to interfere with nociception (77). However, circuitry mediating this effect has been considered from a sensory perspective, and whether pain-modulating circuitry is engaged has not been investigated.

As shown in these examples, top-down inputs can fine-tune the activity of RVM pain-modulating neurons, allowing an organism to adjust sensitivity to potentially painful stimuli depending on other behavioral and physiological priorities.

## “Bottom-Up” Inputs

The burst and pause that define ON- and OFF-cells are responses to “bottom-up” sensory inputs. This rapid “switch” in RVM activity is closely linked to the execution of nocifensive withdrawals that limit or prevent serious injury but has further value as a short-term (seconds to minutes) positive feedback loop reducing the threshold for responding to subsequent stimuli in a potentially dangerous environment (17, 19). Further, sensitization of ON- and OFF-cells, so that the burst and pause are evoked during innocuous stimulation, contributes to allodynia and hyperalgesia in persistent inflammation and following nerve injury (29, 30, 33, 54, 78–80).

A relay through the parabrachial complex (PB) to RVM contributes to both the ON-cell pause and OFF-cell burst. PB is the primary supraspinal target of nociceptive transmission neurons in the superficial dorsal horn (81–83) and projects to the RVM as well as to the PAG and amygdala (84–87). Both cell classes respond to optogenetic stimulation of local parabrachial terminals at short latency, indicating that both receive direct input from PB (88). Optogenetic inhibition of PB terminals in the RVM attenuates ON- and OFF-cell responses to acute noxious stimuli (88). PB also conveys information from inflamed tissue to the RVM, contributing to sensitization of ON- and OFF-cells and hyperalgesia (89). Interestingly, while information related to an acute noxious stimulus or inflammation is relayed through the PB *contralateral* to the inflamed site, information about chronic inflammation is relayed through the PB *ipsilateral* to the inflammation (89).

Other potential sources of nociceptive input to the RVM include direct spinoreticular projections, although these have been considered sparse (90, 91). Relays through higher structures such as the amygdala or insula are also likely, although these higher structures are not required under basal conditions (60, 92, 93).

## Adaptation and Latent Sensitization

As reviewed above, acute injury triggers positive feedback mediated by the recruitment of ON-cells and suppression of OFF-cell firing. This circuit helps establish behavioral hyperalgesia and can be considered protective against further tissue damage. However, a lowered threshold for triggering the ON-cell burst and OFF-cell pause cannot be the whole story. The net influence of the RVM may be time-dependent and reflect a combination of the lowered threshold for evoked responses (i.e., sensitization) and the balance of ongoing activity in the two classes. The latter may serve to limit hyperalgesia (23, 54). For example, in the Complete Freund's adjuvant (CFA) model of inflammatory pain, CFA administration triggers a shift to a prolonged (hours) period of ON-cell firing and OFF-cell suppression. And as would be expected, blocking the RVM within this period early in developing inflammation reverses hyperalgesia in the inflamed paw. However, over subsequent days, this ongoing firing returns to a more normal pattern of alternation, with periods of ON-cell and OFF-cell activity, although the neurons remain sensitized to stimulation of the inflamed paw. Silencing the RVM in fully developed inflammation does not block hyperalgesia and in fact, can enhance hyperalgesia (54). Similar apparently contradictory effects have also been reported after nerve injury. Comparing animals that did and did not develop allodynia after spinal nerve ligation, De Felice and colleagues (23) reported that inactivating the RVM reversed hyperalgesia in the subset of animals that displayed allodynia, but precipitated allodynia and conditioned place aversion in those animals that had not developed behavioral allodynia. This implies that an aberrant nociceptive transmission system can be masked by descending control, preventing the emergence of a pathological pain state. These findings also suggest that understanding the modulatory influence of the RVM requires consideration of ongoing “tone” as well as evoked responses.

The idea that descending control can simultaneously promote and suppress hypersensitivity is particularly compelling in the context of “latent sensitization” (94). This refers to the fact that pain behaviors frequently resolve following an injury, yet animals demonstrate greater susceptibility to a pain response upon subsequent injury or stress (95). Administration of opioid antagonists or blocking of descending inhibition reveals hyperalgesia in animals that have apparently recovered revealing latent sensitization (96, 97). This suggests that the restored balance between ON- and OFF-cell output described above (54) masks the fact that nociceptive transmission remains sensitized. When this compensatory inhibitory system fails, pathological pain results.

## Discussion

The sensation of pain is subjective, and unique to a given individual in a specific context. This is because the sensory experience of pain depends not only on the input of noxious information (nociception), but on the state of the body in a biological, psychological, and social milieu. The brainstem pain-modulating system provides a dedicated circuit through which “bottom-up” sensory inputs and “top-down” cognitive and emotional factors can adjust the threshold and gain for nociceptive transmission.

The output of the best-studied modulatory system is through the RVM. The bidirectional modulatory effects exerted by two RVM cell populations, “ON-cells” and “OFF-cells” are now well documented. However, important open questions remain. One is whether there is a molecular “marker” that defines each cell class. The use of optogenetic manipulation of molecularly defined populations is increasingly popular but should be interpreted with appropriate caution. The fact that activation of a molecularly defined population evokes a particular behavior, or that suppression of such a population interferes with this behavior, is frequently interpreted to mean that this population as a whole is responsible for the behavior. However, without evidence that a molecularly defined population is *functionally* coherent, this is an overinterpretation. Without additional evidence, a more correct conclusion would be that at least some subset of the population contributes to the behavior. A second key open question is exactly how the output from the RVM interfaces with the complex nociceptive circuitry within the dorsal horn. This is certainly an important opportunity for further understanding, and will likely advance in parallel with increasingly sophisticated analyses of dorsal horn circuits.

Finally, efforts to understand top-down inputs to the RVM are starting to bear fruit. The analysis of how intense and mild stress respectively recruit OFF-cells and ON-cells to produce analgesia and hyperalgesia provides a model for teasing out the influence of other cognitive and emotional factors.

In sum, both bottom-up and top-down inputs can influence the output from the RVM, modulating nociceptive transmission, and hence pain. Understanding the interaction between these inputs and defined RVM cell classes will continue to elucidate central mechanisms of pain modulation. This may ultimately make it possible to use these inputs to engage this system, treating clinically relevant pathological pain with fewer side effects.

## Author Contributions

QC and MH wrote the paper. Both authors contributed to the article and approved the submitted version.

## Funding

MH is supported by grants from the NIH (NS098660 and NS120486).

## Conflict of Interest

The authors declare that the research was conducted in the absence of any commercial or financial relationships that could be construed as a potential conflict of interest.

## Publisher's Note

All claims expressed in this article are solely those of the authors and do not necessarily represent those of their affiliated organizations, or those of the publisher, the editors and the reviewers. Any product that may be evaluated in this article, or claim that may be made by its manufacturer, is not guaranteed or endorsed by the publisher.
